# P134: Hand washing: a critical measure in prevention and infection control

**DOI:** 10.1186/2047-2994-2-S1-P134

**Published:** 2013-06-20

**Authors:** R Sharma, M Sharma, V Koushal

**Affiliations:** 1 Hospital Asministration, GMCH 32, Chandigarh, India; 2 Public Health, Chandigarh, India; 3 Hospital Asministration, PGIMER, Chandigarh, India

## Introduction

Hand hygiene is the single most important strategy to prevent HAIs. With the emergence of antibiotic-resistant organisms, the importance of hand hygiene within hospitals has re-emerged as a priority for the 21st century hospital administrators.

## Objectives

The present cross sectional study was conducted in ICUs to assess the hand washing practices being followed among ICU health care workers and factors that motivate or inhibit hand washing.

## Methods

Adherence to hand washing was assessed using three methods i.e. Direct observation, Product utilization and Survey method, updated by Joint Commission (JCI).

## Results

During two week analysis, 2400 hand washing opportunities were observed. Hand washing adherence rate was 86.0%, with highest compliance among nurses (94.0%). Compliance was (95.0%) after patient contact than 72.5% before contact. More than 90.0% staff was aware about facts viz. diseases prevented by hand washing (96.2%), ideal duration of hand washing (92.6%), reduction of HAI with hand washing (98.0%) etc. Reasons for non-adherence emerged as work pressure (94.2%) and unavailability of materials (82.4%).

## Conclusion

The level of compliance (86%) is below the need to be there in ICU otherwise. Easy access to hand-rub solutions, adherence measurement and institutional commitment might contribute to staff sensitivity to hand hygiene practices.

## Disclosure of interest

None declared

